# Microwave propagation and absorption and its thermo-mechanical consequences in heterogeneous rocks

**DOI:** 10.1016/j.minpro.2015.01.003

**Published:** 2015-02-10

**Authors:** R. Meisels, M. Toifl, P. Hartlieb, F. Kuchar, T. Antretter

**Affiliations:** a Montanuniversitaet Leoben, A-8700 Leoben, Franz-Josef-Strasse 18, Austria; b Institute of Physics; c Institute of Mechanics; d Chair of Mining Engineering and Mineral Economics

**Keywords:** Microwave heating, Rock damage, Microwave analysis, Thermally induced stresses, Microwave irradiation experiments

## Abstract

A numerical analysis in a two-component model rock is presented including the propagation and absorption of a microwave beam as well as the microwave-induced temperature and stress distributions in a consistent way. The analyses are two-dimensional and consider absorbing inclusions (discs) in a non-absorbing matrix representing the model of a heterogeneous rock. The microwave analysis (finite difference time domain — FDTD) is performed with values of the dielectric permittivity typical for hard rocks. Reflections at the discs/matrix interfaces and absorption in the discs lead to diffuse scattering with up to 20% changes of the intensity in the main beam compared to a homogeneous model rock. The subsequent thermo-mechanical finite element (FE) analysis indicates that the stresses become large enough to initiate damage. The results are supported by preliminary experiments on hard rock performed at 2.45 GHz.

## Highlights

• We present consistent analyses from microwave propagation to stress distribution.
• The results of the numerical analyses demonstrate the role of the rock microstructure.
• Temperature and stress distributions show favorable conditions for damage initiation.
• Numerical results are backed up by high-power microwave irradiation experiments.
• Implications for applications of microwave-induced rock fragmentation are drawn.

## 1 Introduction

The fragmentation of rocks is a process which is applied e.g. for excavation of mines, tunneling, extraction of valuable ores and processing of pre-conditioned rocks. Comminution consumes roughly 29% of the total energy spent for mining in the USA (DOE, 2005) and amounts up to 2% of the total energy used in several mining countries like USA, Australia and South Africa (Tromans, 2008). Only less than 1% of this energy is actually used for the generation of new surfaces whereas the rest is turned into noise and heat (Fuerstenau and Abouzeid, 2002, DOE, 2007). Many of the techniques applied use mechanical cutting tools. Techniques assisting these tools have a high potential for reducing the energy consumption. One of them is the application of high-power microwaves prior to the mechanical treatment. The purpose is to heat the rock in a way that large temperature gradients are established that generate thermally induced stresses which should exceed the strength of the material. The final goal is to develop economical methods assisting mechanical tools for breakage, cutting, and comminution. The investigations prior to the late 1980s and up to 1999 are reviewed by Santamarina (1989) and Haque (1999), respectively. Laboratory tests mainly concerning comminution have been performed in multi-mode microwave ovens (e.g. Walkiewicz et al., 1991, Kingman and Rowson, 1998, Amankwah et al., 2005, Peinsitt et al., 2010, Hartlieb et al., 2012, Hassani et al., 2012) and in a single-mode cavity (Kingman et al., 2004). Substantial weakening of the rocks has been observed whereupon the energy consumption was significantly lower in the single-mode cavity. Hard-rock drilling was investigated by Lindroth et al. (1993); microwave irradiation increased the drilling rate by a factor of up to 6.5 at the highest temperatures reached. Jerby et al. (2002) reported a microwave drill for non-conductive materials, where 2 mm diameter, 2 cm deep holes were drilled within less than a minute.

The response of rocks and minerals to microwaves including their heating by the absorption of the microwaves has been investigated during the past 50 years. Despite this long period no large-scale application or commercial equipment exists. To our opinion this is partly due to the lack of basic understanding of the processes involved. A general property of rocks which should be taken into account is their multi-phase constitution — grains of different shapes and compositions being distributed in a matrix. In the case of irradiation of a large block or of a tunnel face the microwave is applied as a beam in order to achieve a high power density. High power density is maintained in the rock at least within the penetration depth of the microwave. An unavoidable effect is the Gaussian broadening of the beam which is significant if the width of the beam is not much larger than the wavelength. In heterogeneous materials, additionally, diffuse scattering can cause deviations from Gaussian-beam propagation.

The absorption of microwave radiation in a rock depends on the high-frequency dielectric properties of its constituents. More strongly absorbing constituents are selectively heated leading to temperature gradients and different volumetric expansion. As a consequence internal compressive and shear stresses are expected to build up causing cracks, which are responsible for a reduction of rock strength consequently leading to a reduction of mechanical energy required for fragmentation of the rock mass or even to a fragmentation solely due to the microwave absorption. First simulations performed by Jones et al. (2005) corroborate that the microstructure has a high influence on strength reduction due to selective heating. Wang et al. (2008) as well as Ali and Bradshaw, 2009, Ali and Bradshaw, 2010, Ali and Bradshaw, 2011 discuss the necessity of a minimum microwave power density necessary for successful generation of cracks which cannot be compensated by longer irradiation times. This scenario has not been met by most of the experiments performed since the exposure time to the microwave power is long compared to the time needed for heat conduction between the grains of the rock (in the following called “slow heating”). As a consequence the temperature gradients do not only appear on the length scale of the microstructure of the rock. They mainly appear on the length scale of the penetration depth and the beam diameter in the cases where these are smaller than the sample size. If the heating is homogeneous in the whole rock temperature gradients appear only due to heat transfer to the surrounding medium and are on the length scale of the sample.

The dielectric properties of a solid are described by the complex dielectric constant (permittivity): *ε* = *ε*_*r*_ + *iε*_*i*_ = *ε*_0_(*κ*_*r*_ + *iκ*_*i*_). *κ*_*r*_ is the real part, *κ*_*i*_ the imaginary part of the relative permittivity *κ*, and *ε*_0_ is the permittivity of vacuum. At microwave frequencies the *κ*_*r*_ values of most rocks are between 2 and 10, the *κ*_*i*_ values between 10^− 3^ and 50; both depend on frequency and temperature (Santamarina, 1989). For *κ*_*i*_ < *κ*_*r*_ the reflection is mainly determined by *κ*_*r*_, the absorption or losses by *κ*_*i*_. A useful quantity for characterizing the absorption is the penetration depth *D*_*p*_. It can be defined via the decrease of the microwave power *P* with depth *z* in a material: *P* = *P*_0_*exp*[− *z*/*D*_*p*_] (another definition uses the electric field strength *E* ~ *P*^1/2^). 67% of the power is absorbed within the penetration depth. The penetration depth *D*_*p*_ (= inverse of the absorption coefficient) depends mainly on *κ*_*i*_. Penetration depths vary from millimeters to meters. As an example at 2.45 GHz, a widely used frequency, for *κ*_*r*_ ≈ 10 the penetration depth *D*_*p*_ is about 5 m with *κ*_*i*_ ≈ 0.01 and 0.01 m with *κ*_*i*_ ≈ 5. The *κ*_*i*_ range useful for heating of rocks is approximately 0.1–2 corresponding to *D*_*p*_ ≈ 50–2 cm. Strong microwave absorbers are, e.g., magnetite, chalcopyrite and water, whereas poor microwave absorbers are feldspar, quartz, marble and ice. *κ*_*i*_ varies with the content of water and salt. Metallic constituents in a rock reflect microwaves into the non-metallic constituents.

The goal of the present work is to study the influence of the heterogeneity on the propagation of a microwave beam in a two-component rock, i.e. the amount of diffuse scattering as well as the heating and the formation of microwave induced stresses. This is done in a comprehensive analysis chain starting from the numerical solution of Maxwell's equations by means of a finite difference scheme (finite difference time domain — FDTD, see Yee (1966) and Taflove (1995)), a subsequent heat transfer calculation and concluding with a mechanical analysis of the concomitant stresses by means of the finite element method (FEM). Results of such a calculation for small cylindrical samples fully irradiated by microwaves were reported previously (Hartlieb et al., 2012). They are representative for samples in a microwave oven as used in many experimental studies. In the present work, a situation closer to a large-scale experiment is considered, i.e. the dimensions of the model rock are larger than the width of the microwave beam and of the order of the penetration depth of the microwaves, resulting in a different temperature field and thus thermal stresses that have not been considered in Hartlieb et al. (2012) and Jones et al. (2007). The entire simulation chain presented here for a partially irradiated mass of rock has, to the authors' knowledge, not yet been reported in the literature.

## 2 Model for a heterogeneous rock and the wave propagation

**Fig. 1:**
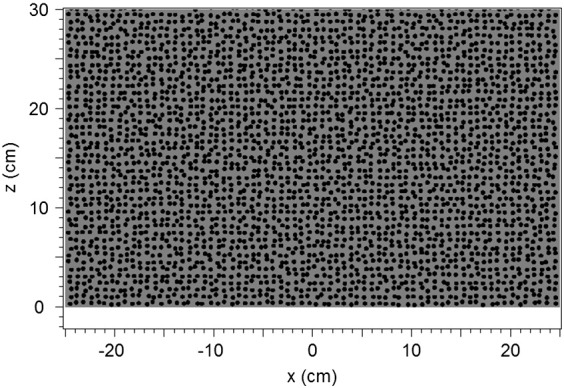
Two-dimensional model of a block of rock with statistical distribution of discs in a matrix (details see Fig. 2). The microwave source (not shown) is positioned at x = 0 and z = − 1 cm. Its width in x direction is 8.6 cm.

**Fig. 2:**
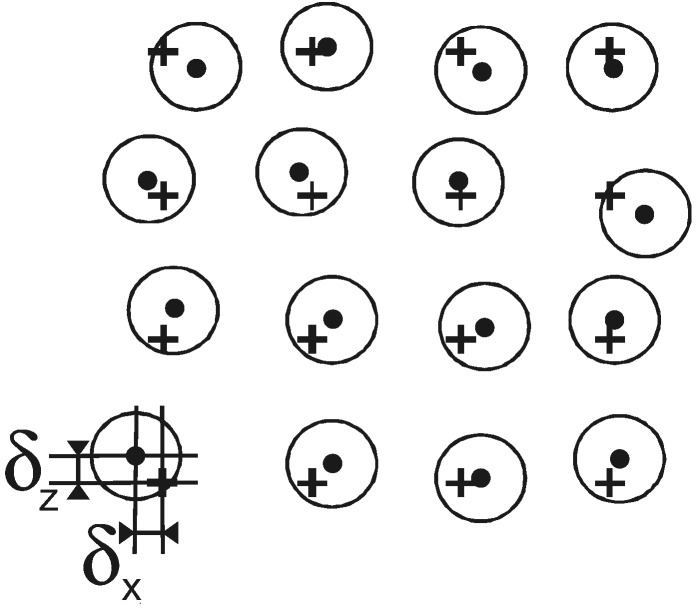
Enlarged section of the two-dimensional model rock. The grains in the matrix are represented by discs positioned on a square lattice with positional disorder. The crosses represent the lattice points, the dots the actual centers of the discs. *δ*_*x*_ and *δ*_*z*_ denote the deviation from the ideal position on a lattice point. After Meisels and Kuchar (2007).

As an approach to a heterogeneous rock with grains in a matrix we use a two-dimensional two-component model with circular discs distributed randomly in a matrix (Fig. 1). The geometrical configuration was generated by means of a user code written in C following the procedure described by Meisels and Kuchar (2007) where the random distribution is realized by positional disorder of the discs on a square lattice. The strength of the positional disorder is determined by the parameter *δ*. The deviation of the *i*-th element from the ideal position on a square lattice in the *x* and *z* directions, *δ*_*x*,*i*_ and *δ*_*z*,*i*_, is limited by *δ* (*δ*_*x*,*i*_, *δ*_*z*,*i*_ < *δ*) and has a uniform distribution (Fig. 2). In the calculations we choose *δ* to be 25% of the lattice constant *a* = 0.71 cm. The diameter of the discs is 0.467 cm corresponding to a filling factor *f*, i.e., the fraction of the area occupied by the discs relative to the total area, of 0.34. These values (parameters summarized in Table 1) mean that the arrangement of the discs is far away from percolation but that some of the discs can overlap by about 0.1 cm. This model is simplified but should qualitatively give a good estimate of the effects of scattering due to differences in the dielectric properties of inclusions and matrix in a rock. The generated input file containing the geometry information can be imported to both the FDTD solver (RSoft, 2014) as well as the finite element program (ABAQUS, 2012). The model – being two-dimensional and for a two-component rock – can be extended to three dimensions, more than two constituents, and different shapes of the constituents by using the same kind of script.

The effect of the disorder on the microwave propagation in the two-dimensional model rock is calculated by using the FDTD method (Yee, 1966). We calculate the distributions of the squared electric field *E*_*y*_^2^, which is a measure of the microwave energy density, and of the absorbed power density. The knowledge of the distributions of *E*_*y*_^2^ and the absorption is the basis for subsequent calculations of temperature distributions, thermal gradients and stresses.

The density distribution of the absorbed power is directly interpreted as heat sources entering the heat conduction equation which is solved numerically with a commercial finite element package ABAQUS (2012). The resulting temperature distribution will show a speckle pattern corresponding to the heterogeneous distribution of heat sources. Given the inhomogeneous thermo-elastic properties of the rock material (disc and matrix) and using the time varying temperature field as thermal load, a subsequent stress analysis allows working out the major principal stresses as indicators for damage initiation in brittle materials. Results of the calculation of the stresses gained by the inhomogeneous material distribution will be given for typical hard rock properties. The conditions are chosen so that they correspond to “slow heating” defined in Section 1.

As the microwave frequency a standard industrial frequency, 2.45 GHz, is chosen. The wavelength in a rock with, e.g., *κ*_*r*_ = 7 is approximately 5 cm. The size of the rectangular model rock is 50 cm in width and 30 cm in length (direction of microwave propagation), the size of the computational domain is 50 · 32 cm^2^ as shown by the black frame in Fig. 1. The computational grid has a lattice constant of 0.05 cm. To find out whether this grid is sufficiently fine a calculation was performed with half the value (0.025 cm) for the situation of Fig. 6(a) (see Section 4). The results for *E*_*y*_^2^ along *x* = 0 cm deviate by less than 2 · 10^− 4^ in the units of Fig. 6(a). This means about 0.1% relative difference of the results for 0.05 cm from those for 0.025. Therefore, we consider the value of 0.05 cm as sufficiently small for obtaining reliable results.

**Fig. 3:**
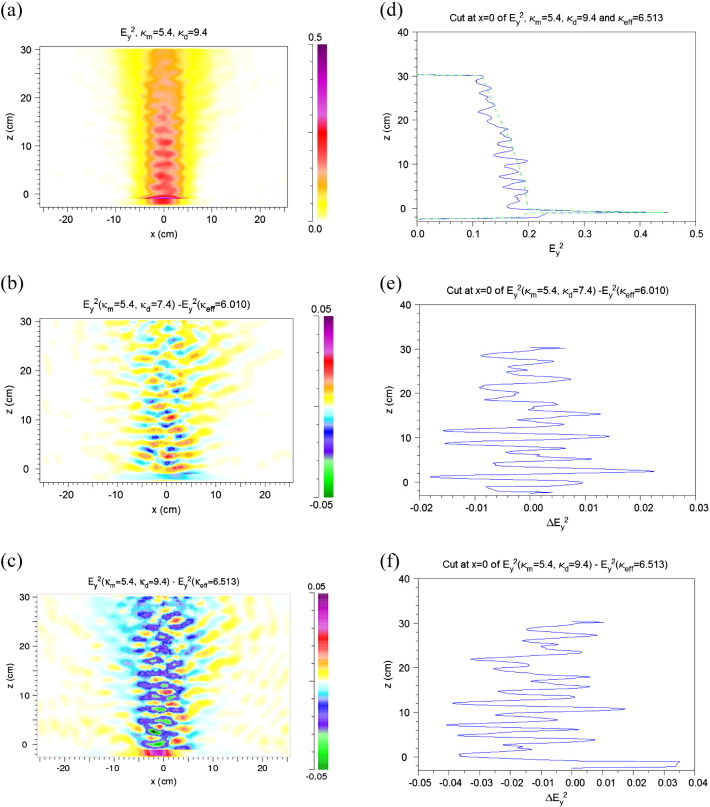
Results for heterogeneity in the real parts of the permittivity and no loss (*κ*_*m*.*i*_ = *κ*_*d*,*i*_ = 0). (a) Distribution of *E*_*y*_^2^ in the model rock and in front of it. *κ*_*m*_ = 5.4, *κ*_*d*_ = 9.4. Definition of *E*_*y*_^2^ see Section 2. (b, c) Difference Δ*E*_*y*_^2^ of the distributions of *E*_*y*_^2^ in the heterogeneous and homogeneous model. *κ*_*m*_ = 5.4, *κ*_*d*_ = 7.4 and 9.4, respectively. *κ*_*eff*_ calculated from Eq. (4). (d–f) Cuts along the z direction at x = 0 through the *E*_*y*_^2^ and Δ*E*_*y*_^2^ distributions of (a), (b), and (c). In (d) the solid line is for the heterogeneous model, the dashed one for the homogeneous model.

The microwave source with width 8.6 cm (corresponding to the opening of the waveguide for 2.45 GHz) is positioned 1 cm in front of the sample (Fig. 1, *x* = 0, *z* = − 1 cm). It emits a beam in positive z direction with the polarization in y direction and the value of the time-averaged *E*_*y*_^2^ being = 1 (at *x* = 0, in dimension-less units). The shape of the beam is Gaussian (Kogelnik and Li, 1966) which means that the width in x direction increases in the propagation direction as visible e.g. in Fig. 3(a). Part of the beam is reflected at the air/rock interface and at the discs/matrix interfaces in the interior which interferes with the emitted beam. At the boundary of the computational domain the radiation is totally absorbed. This ensures that the wave pattern remains unaffected by reflections from these boundaries and that the effect of the disorder can be clearly observed. Also for a real environment – e.g. excavation of a mine or tunneling operation – the model with absorbing boundary conditions is relevant because there the thickness would be practically infinite compared to the penetration depth.

In the figures shown in 4, 4.1, 4.2, 4.3, *E*_*y*_^2^, time-averaged over the 24th period of the microwave is displayed. The 24th period is chosen since the *E*_*y*_^2^ pattern in the whole model rock is stable after this time. The method for calculating temperature and stress distributions – a finite element analysis – is described in Section 4.5.

## 3 Material parameters

### 3.1 Dielectric properties

The values of the real and imaginary parts of the permittivity used in the model calculation are chosen to be in the range given for rocks in the literature. Typical values at room temperature and a microwave frequency of 3 GHz are those of basalt and granite which vary over a wide range, viz. for *κ*_*r*_ from 5.4 to 9.4 (basalt) and 5 to 5.8 (granite) and for *κ*_*i*_ from 0.08 to 0.88 (basalt) and 0.03 to 0.2 (granite) (Santamarina, 1989). Since real rocks consist of several constituents these values are effective values *κ*_*eff*_. With these effective values we calculate the propagation in a homogeneous model for comparison with our heterogeneous two-component model. For the latter one the individual permittivities of the constituents are determined so that they yield the effective values. For this purpose we use Bruggeman's effective medium theory (Bruggeman, 1935). For the case of circular discs in a matrix the relation between the individual permittivities and the effective one is given in the following equations. The permittivities can be complex which is the case for the situations considered in 4.2, 4.3.

(1) $$f\frac{\kappa_{d}-\kappa_{\mathrm{eff}}}{\kappa_{d}+\kappa_{\mathrm{eff}}}+\left(1-f\right)\frac{\kappa_{m}-\kappa_{\mathrm{eff}}}{\kappa_{m}+\kappa_{\mathrm{eff}}}=0$$

(2) $$\kappa_{m}=\frac{\left(2f-1\right)\kappa_{d}-\kappa_{\mathrm{eff}}}{\left(2f-1\right)\kappa_{\mathrm{eff}}-\kappa_{d}}\kappa_{\mathrm{eff}}$$

(3) $$\kappa_{d}=\frac{\left(1-2f\right)\kappa_{m}-\kappa_{\mathrm{eff}}}{\left(1-2f\right)\kappa_{\mathrm{eff}}-\kappa_{m}}\kappa_{\mathrm{eff}}$$

(4) $$\kappa_{\mathrm{eff}}=\left(1/2-f\right)\left(\kappa_{d}-\kappa_{m}\right)+\sqrt{{\left(1/2-f\right)}^{2}{\left(\kappa_{d}-\kappa_{m}\right)}^{2}+\kappa_{d}\kappa_{m}}\text{.}$$

*f* is the filling factor defined in Section 2. For the calculation of the *E*_*y*_^2^ distribution, depending on the situation considered, either values for *κ*_*r*_ and *κ*_*i*_ of the matrix and discs are chosen and *κ*_*eff*_ is calculated, or values for *κ*_*r*_ and *κ*_*i*_ of the matrix and *κ*_*eff*_ are chosen and *κ*_*r*_ and *κ*_*i*_ of the discs are calculated. In the first three sections of Section 4 we will show results for different *κ*_*r*_ and *κ*_*i*_ values for the homogeneous and the heterogeneous cases as well as the difference between the two. This procedure allows deducing the effect of the heterogeneity of the rock via the strength of the diffuse scattering. The heterogeneity is due to the discontinuities of the real parts of the permittivity at the discs/matrix interfaces and due to the non-zero imaginary part in the discs. The reflections at the interfaces are governed by the Fresnel formula for the reflection coefficient *r*. For normal incidence *r* is given by [(*n* − 1)^2^ + *k*^2^]/[(*n* + 1)^2^ + *k*^2^]. For *κ*_*i*_ ≪ *κ*_*r*_ the refractive index *n* is proportional to *κ*_*r*_ and the extinction coefficient *k* (~ absorption coefficient) proportional to *κ*_*i*_. In order to find out whether the real or the imaginary part is predominantly responsible for diffuse scattering, we consider three cases of the two-component model (4.1, 4.2, 4.3): matrix (*m*) and discs (*d*) differ (a) only in the real part (with the imaginary parts being zero), (b) only in the imaginary part, (c) both in the real and the imaginary part of the permittivity. In all cases the permittivity of the matrix is assumed to be real, i.e. no loss of radiation or absorption occurs there.

### 3.2 Thermo-mechanical properties

The temperature dependent thermo-mechanical material data (density, heat capacity, thermal conductivity, thermal expansion, Young's modulus, Poisson's ratio) of the two components (disc and matrix) used in the model are given in Table 2. Here we assume that the discs have the properties of plagioclase (Ab_56_An_44_) and the matrix those of quartz being constituents of hard rocks like granite.

The thermal and mechanical properties of quartz suddenly change at 573 °C due a phase transformation. In the current study, however, this will have no effect, since the calculated temperatures stay below the transformation temperature.

## 4 Results

Results for the distributions of the square of the electric field, *E*_*y*_^2^, are shown in 4.1, 4.2, 4.3, for the absorption in Section 4.4, and the temperature and stresses in Section 4.5.

### 4.1 E_y_^2^, loss-less case, κ_m,r_ ≠ κ_d,r_, κ_m,i_ = κ_d,i_ = 0

The input values are those for *κ*_*m*,*r*_ and *κ*_*d*,*r*_. They are given in Fig. 3 as well as the effective value calculated according to Eq. (4). The imaginary parts are zero. Under this assumption only reflections at the interfaces of the matrix and the discs, but no absorption occurs.

In Fig. 3(a) an example of the distribution of *E*_*y*_^2^ in the block of rock in our heterogeneous two-component model is shown. A stripe-like pattern occurs which does not exist in the homogeneous case. The *E*_*y*_^2^ distribution in the homogeneous case corresponds to that of a Gaussian beam. More detailed and weaker deviations from the distribution in the homogeneous case are hardly visible in such a presentation. However, they can be made clearly visible in the difference Δ*E*_*y*_^2^ between the results of the heterogeneous and homogeneous models, Δ*E*_*y*_^2^ = *E*_*y*_^2^(*κ*_*m*_,*κ*_*d*_) − *E*_*y*_^2^(*κ*_*eff*_), as shown for two different ratios of the permittivities of the matrix and the discs in Fig. 3(b–c). Cuts along the z direction through the *E*_*y*_^2^ and Δ*E*_*y*_^2^ distributions at x = 0 cm are shown in Fig. 3(d–f).

The difference distributions show that the deviations of the heterogeneous from the homogeneous results are up to 20% in the main beam. Due to scattering at the discs, energy density also occurs far outside the main beam being, however, very low.

### 4.2 E_y_^2^, lossy case, κ_m,r_ = κ_d,r_, κ_m,i_ = 0,κ_d,i_ ≠ 0

**Fig. 4:**
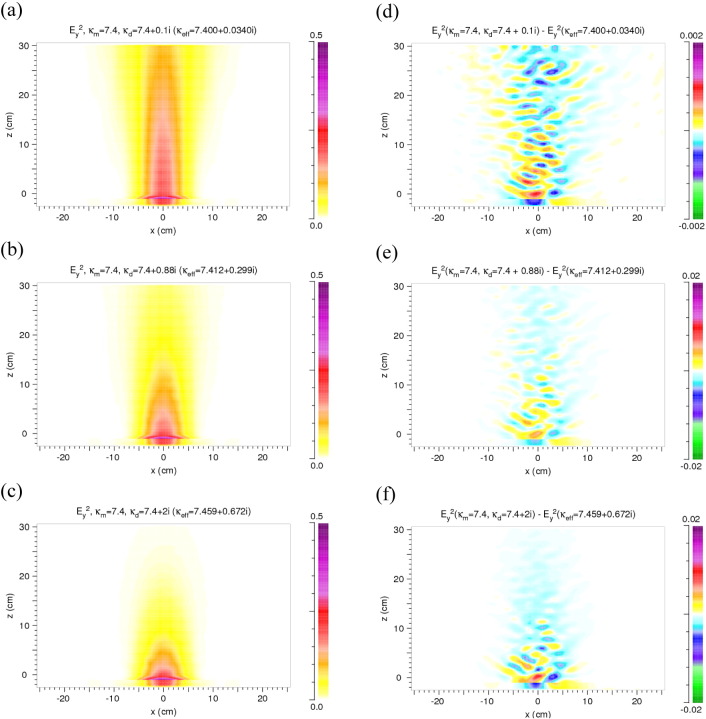
Results for heterogeneity only due to absorption in the discs: *κ*_*m*,*r*_ = *κ*_*d*,*r*_ = 7.4, *κ*_*d*,*i*_ = 0.1, 0.88 and 2.0, *κ*_*m*,*i*_ = 0. (a–c) Distribution of *E*_*y*_^2^ in the model rock and in front of it. (d–f) Difference between the distributions of *E*_*y*_^2^ in the heterogeneous and homogeneous models. Note the change of the color scale.

In this case deviations from the homogeneous results can originate only from *κ*_*d*,*i*_ being ≠ 0. *κ*_*d*,*i*_ ≠ 0 causes absorption in the discs but also weak reflection at the discs/matrix interfaces. *κ*_*m*,*r*_ and *κ*_*d*,*r*_ are equal and chosen to be 7.4. *κ*_*d*,*i*_ is varied, *κ*_*eff*_ is calculated from Eq. (4). The results are shown in Fig. 4. Compared to Fig. 3(a) the stripe like structure is extremely weak here and not visible on the color scale used; also the deviations from the homogeneous case are weaker in the range of the main beam and less energy is scattered sideward.

### 4.3 E_y_^2^, lossy case, κ_m,r_ ≠ κ_d,r_, κ_m,i_ = 0,κ_d,i_ ≠ 0

**Fig. 5:**
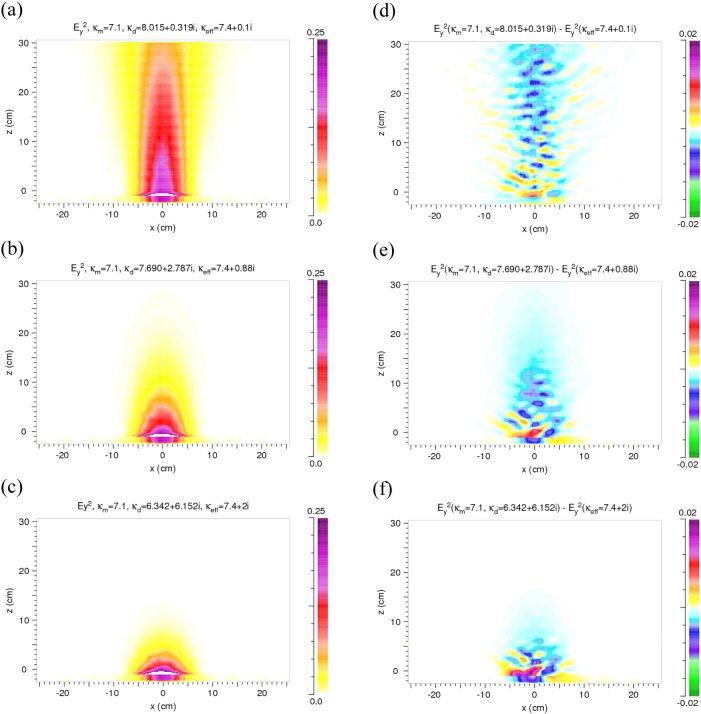
Results for differences in the real as well as in the imaginary part of the permittivity of the matrix and the discs, i.e. heterogeneity due to reflection at the discs/matrix interfaces and absorption in the discs. (a–c) Distributions of *E*_*y*_^2^ in the model rock and in front of it. (d–f) Distributions of Δ*E*_*y*_^2^. Permittivity values: *κ*_*m*,*r*_ = 7.1, *κ*_*m*,*i*_ = 0, and *κ*_*eff*,*r*_ = 7.4. Values of *κ*_*eff*,*i*_ (varied), and of *κ*_*d*,*r*_ and *κ*_*d*,*i*_ (calculated from the other values, Eq. (3)) are given in the figures.

**Fig. 6:**
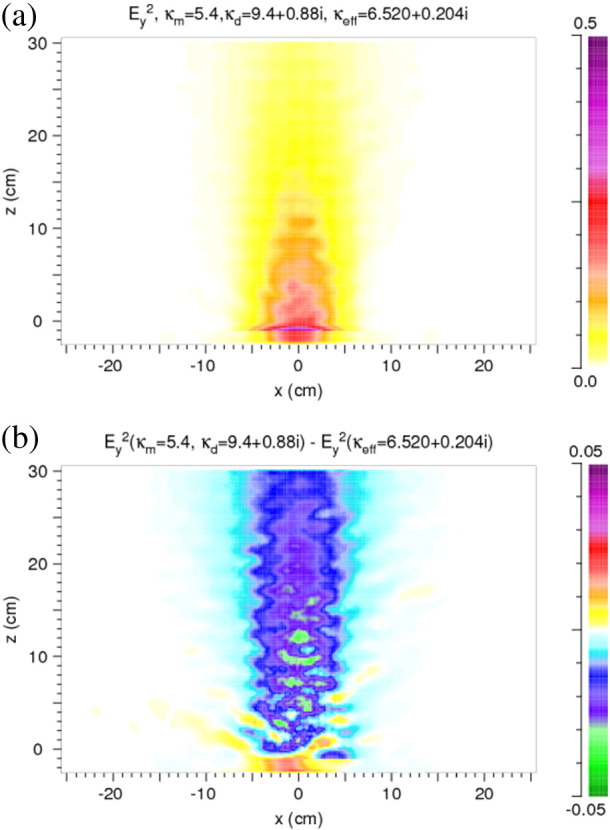
*E*_*y*_^2^ (a) and Δ*E*_*y*_^2^ (b) for a larger difference of the real parts (*κ*_*m*,*r*_ = 5.4, and *κ*_*d*,*r*_ = 9.4) than in Fig. 5; *κ*_*d*,*i*_ = 0.88. In this case *κ*_*eff*_ is calculated from the other values (Eq. (4)).

This section analyzes the combined effect of differences in *κ*_*m*_ and *κ*_*d*_. The starting point for the calculations of Fig. 5 is typical experimental values of the complex permittivity of rocks, i.e. *κ*_*eff*_ in our numerical analysis. Its real part is chosen to be 7.4 – an average value for basalt – its imaginary part is varied as given in the figures. *κ*_*m*_ is real and set equal to 7.1; *κ*_*d*_ is complex and calculated from Eq. (4). With increasing imaginary part the penetration depth decreases. The strongest positive deviations from the homogeneous case occur near the first air/rock interface. Further away from it the deviations are negative. In order to increase the reflections at the discs/matrix interfaces a larger difference between *κ*_*m*,*r*_ and *κ*_*d*,*r*_ is chosen in Fig. 6. The stripe-like structure is still present but damped due to the absorption, which also strongly reduces the sideward scattering.

Generally, in the main beam the deviations Δ*E*_*y*_^2^ are up to 10% (positive and negative). The sideward scattering is stronger than in Fig. 4 (absorption in the discs only), but weaker than in Fig. 3 (reflections at the discs/matrix interfaces only).

### 4.4 Absorbed power density

**Fig. 7:**
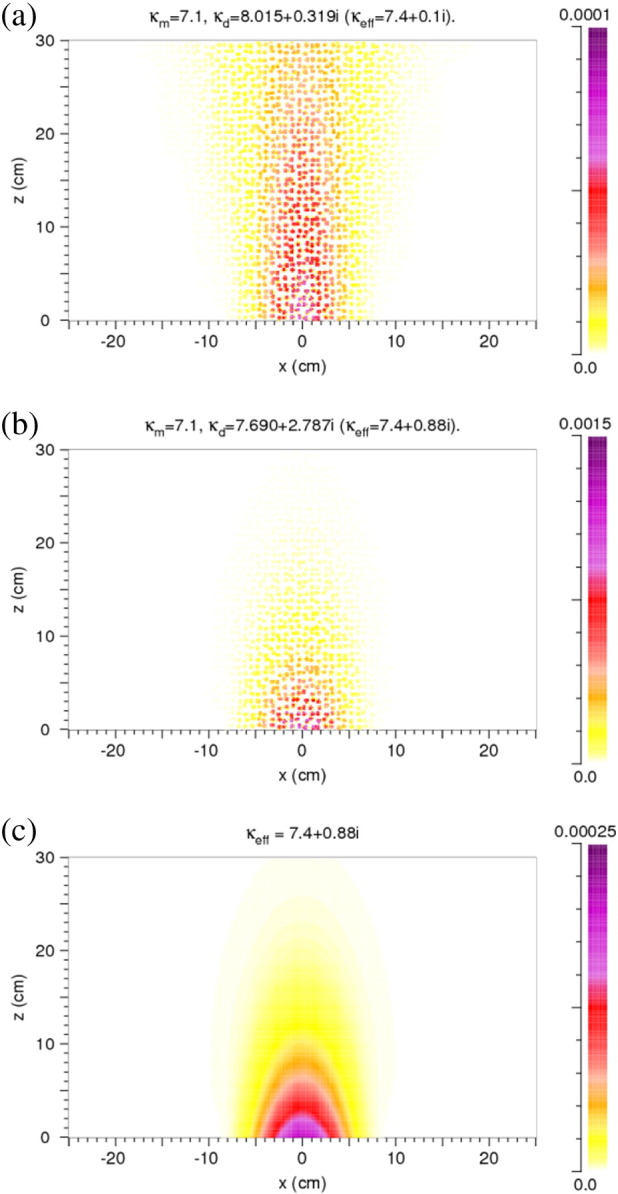
Pattern of the absorbed power density within the model rock. (a) Large penetration depth. (b) Small penetration depth. Compare *E*_*y*_^2^ in Fig. 5(a) and (b). The color scale indicates the time-averaged absorbed power density in units of W/cm^3^ normalized to a time-averaged *E*_*y*_^2^ of 1 V^2^/cm^2^ of the microwave source at x = 0 cm and z = − 1 cm. Note the difference in the values of the absorbed power. (c) Absorbed power density for a homogeneous reference case (cf. (b)).

For a calculation of the heating of a rock the absorbed power density is a necessary input. Therefore, we show examples for two cases of Section 4.3 in Fig. 7. The absorbed power density is defined by *ωε*_*i*_*E*_*y*_^2^. According to the assumption, viz. *κ*_*m*,*i*_ = 0, absorption only occurs in the discs. Its distribution corresponds to the one of *E*_*y*_^2^.

### 4.5 Temperature and stresses

Based on the distribution of the absorbed power density obtained from the two-component FDTD calculation with microwave absorption only at the discs (*κ*_*d*,*i*_ ≠ 0) and a non-absorbing matrix, a finite element (FE) heat transfer analysis ABAQUS (2012) is performed in order to assess the selective heating of the rock sample. The sample parameters are the same as in Fig. 7(b). A 2D-model with a regular mesh according to the FDTD analysis is used. To model the material inhomogeneities in the vicinity of the microwave irradiation spot in an appropriate manner, a finer discretization in this region is used. The microwave energy input is simulated by applying a distributed body heat flux derived from the distribution of the absorbed power density and the duration of the irradiation. The total energy input is obtained in an iterative manner by evaluating the energy balance of the thermal model assuring that the total available microwave power does not exceed the given energy input by the microwave source. This value is chosen according to the microwave power generated by the source (25 kW) and assumed losses of 30% resulting in a power input of 17.5 kW during 15 s irradiation time (conditions of the experiment described below). On the front face (normal vector points in negative z-direction) a thermal conductance of 20 W/m^2^ K and heat transfer coefficient of 0.8 and an ambient temperature of 25 °C is assumed. The initial temperature of all nodes is set to 25 °C.

**Fig. 8:**
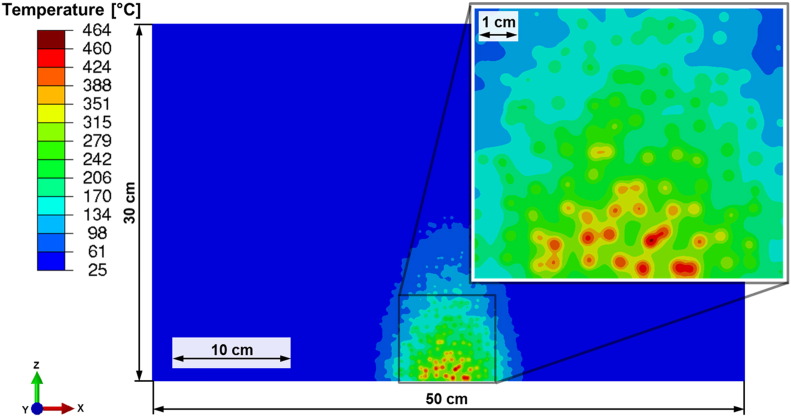
Temperature distribution in °C after 15 s of microwave irradiation (17.5 kW) in the 2D model rock corresponding to Fig. 7(b). Sample size as in Fig. 3, Fig. 4, Fig. 5, Fig. 6, Fig. 7.

The maximum temperature (464 °C) in the rock sample is obtained inside an inclusion (two overlapping discs) in a distance of 1 cm from the front surface (Fig. 8). Furthermore selective heating of the discs due to the selective absorption and variation of the thermal properties can be observed and leads to significant thermal gradients between the two constituents. 30 s after switching off the microwave source the temperature differences between the discs are equalized and a surface temperature of 310 °C is observed agreeing with experimental results discussed below.

With the time varying temperature field as input, a subsequent stress analysis is conducted assuming plane strain conditions and a heterogeneous material as in the microwave analysis. No effect due to a sudden change in volume is expected since the temperature does not exceed 573 °C — the temperature of the transformation from α-quartz to β-quartz. As the material can be considered as brittle, the maximum principal stress is an appropriate measure for assessing damage initiation.

**Fig. 9:**
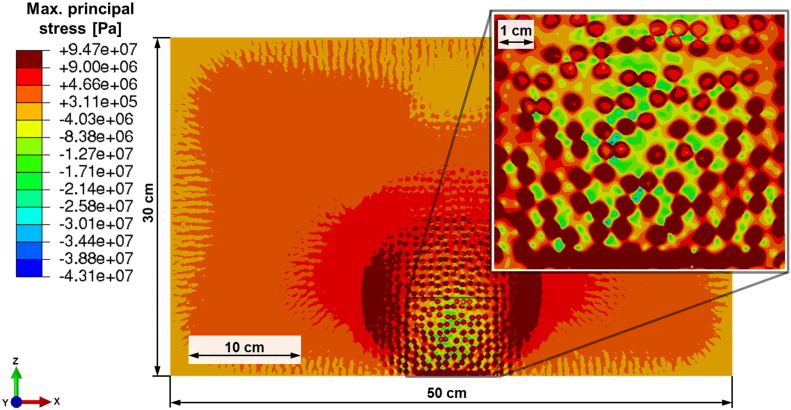
Maximum principal stresses in Pa in the model rock after 15 s of microwave irradiation calculated from the temperature distribution of Fig. 8. Dark red indicates values larger than 9 MPa.

High values of maximum principal stresses are observed in areas on the front face and right and left of the microwave heated region exceeding the tensile strength of a typical rock of about 9 MPa (Hustrulid et al., 2001) (Fig. 9). These stresses are caused by the high thermal gradients. Furthermore critical tensile stresses are obtained in large areas inside the discs particularly along their circumference.

Most of the matrix visualized in the detail of Fig. 9 is under compression due to strong selective heating and significantly higher thermal expansion of the matrix compared to the discs (cf. Table 2). Caused by the high compression stresses the matrix acts as a crack arrest. Therefore it is expected that damage appears just in a small area and the remaining part will contain some subcritical cracks.

**Fig. 10:**
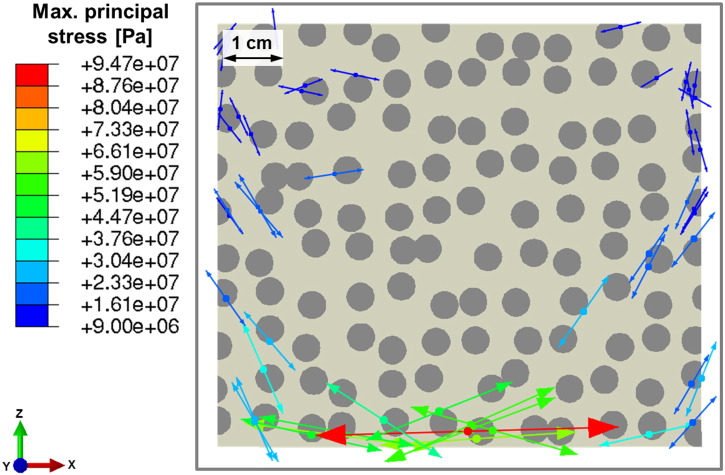
Vector plot of the maximum principal stress (greater than tensile strength of 9 MPa) after 15 s of microwave irradiation in the magnified area shown in Fig. 9; the arrows indicate the direction of the normal to a potential crack plane.

Driven by the high maximum principal stresses near the irradiation spot, cracks may be initiated at the circumference of the discs as well as in the matrix. Most of the cracks are concluded to be in the radial direction of the discs (Fig. 10). Based on the highly loaded matrix in this area, damage propagation will also take place there. Since damage occurs very early during the irradiation, stresses will redistribute once crack propagation is taken into account.

A comparative analysis in which permuted material definition is assumed (discs are composed of quartz and matrix of plagioclase) obtained a different behavior. There high values of maximum principal stresses are observed in large areas inside the matrix. Interface cracks in the matrix may be initiated between the various discs and between inclusions and boundary surface. Initial cracks can potentially grow within the highly loaded matrix entailing severe rock fragmentation.

## 5 Discussion

**Fig. 11:**
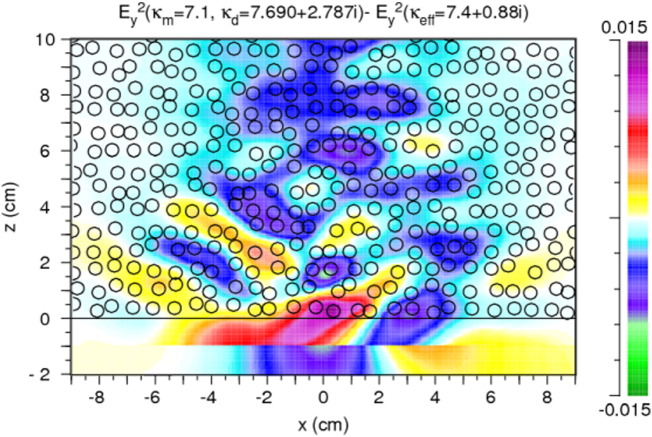
Comparison of disc arrangement (circles) and the Δ*E*_*y*_^2^ distribution (color pattern). Enlarged section of Fig. 5(e) near the microwave source.

**Fig. 12:**
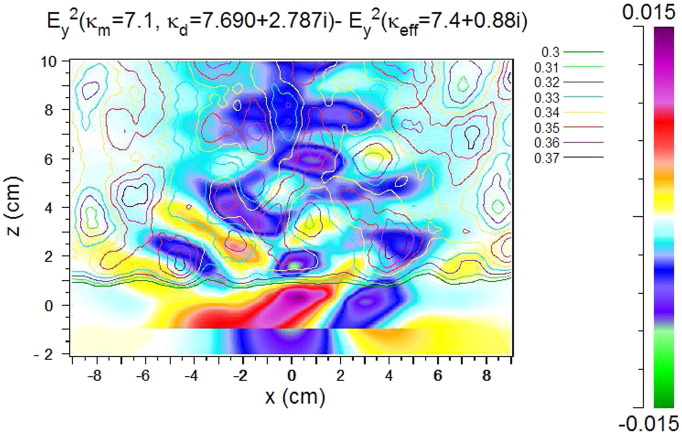
Comparison of density variations of the disc distribution (contour lines) and the Δ*E*_*y*_^2^ distribution as in Fig. 5(e) (color pattern). 0.34 is the average density (= filling factor). Permittivities of discs and matrix same as in Fig. 7. Near the boundary of the model rock (x = 0) the values of the density approach zero. Contours with values smaller than 0.3 are not shown here.

Regarding the results of the microwave analysis, in the heterogeneous cases stripe-like and finer structures are visible in the *E*_*y*_^2^ and the Δ*E*_*y*_^2^ patterns. The stripes are well pronounced in the case of a large difference of *κ*_*m*,*r*_ and *κ*_*d*,*r*_, weaken with increasing absorption in the discs, and almost disappear without a contrast in the real part of the permittivity. In order to identify their origin we compare the pattern with the arrangement of the discs (Fig. 11) and with variations of the density of the discs (Fig. 12). For calculating the variation of the density the disc distribution is smoothed over about 1.5 cm and contours of equal density are drawn. The latter ones are shown for values around the filling factor defined in Section 2 (*f* = 0.34). Neither the disc arrangement nor the density variation shows a correlation with the details of the Δ*E*_*y*_^2^ pattern, with the exception of few coincidences in the very first part (up to 10 cm) of the model rock as visible in Fig. 12 for strong absorption in the discs. In these areas the strong absorption causes negative Δ*E*_*y*_^2^ values (blue/green) at higher density (> 0.34 contours) and positive Δ*E*_*y*_^2^ values (yellow/red) at lower density values (< 0.34 contours). Also, with the strong absorption the stripe-like structure disappears.

The stripe-like structure is not exactly regular. Nevertheless, an average period of the pattern can be determined which is about half of the wavelength in the rock (*λ*_*w*_ ≈ 4–5 cm). It increases with increasing wavelength. This leads to the conclusion that the stripe-like structure is an interference effect dominated by the effective permittivity with the deviations from regularity and the finer structure depending on details of the interference of partial waves between the discs.

The dependence on the difference between *κ*_*m*,*r*_ and *κ*_*d*,*r*_ is best visible in the case of no absorption (Fig. 3). It is interpreted as follows: With the larger difference stronger *E*_*y*_^2^ contributions (yellow) appear further outside the main beam, weaker contributions near its center (blue and green). Due to scattering and interference, however, also spots of increased *E*_*y*_^2^ (red) occur in the central region. Also stronger *E*_*y*_^2^ contributions are reflected into the region at *z* < 0 (red) when the difference is larger. With absorption included (Fig. 5) *E*_*y*_^2^ is increased in the first part of the beam within the rock, however, scattering towards the side and back to the source is weaker due to absorption.

Considering the results of Fig. 3, Fig. 4, Fig. 5 the question arises whether the difference between discs and matrix in the real or in the imaginary part of the permittivity dominates the diffuse scattering. Without absorption the reflection at the discs/matrix interfaces produces relatively large deviations from the homogeneous case and scatters a significant amount of radiation out of the primary beam towards the sides of the model rock. With the difference being present only in the imaginary part, scattering also occurs – but to a lesser extent – in the sideward directions since reflections at the discs/matrix interfaces are weak and the absorption reduces the intensity on the way to the boundary of the rock. The combined effect of the differences in the real and the imaginary parts leads to *E*_*y*_^2^ distributions with relatively strong deviations from the homogeneous case but only in the main beam. The sideward scattering is weak due to the absorption in the discs (Fig. 5).

As results of considering absorption in the discs only, a strong selective heating and therefore high thermal gradients between the constituents are observed (Fig. 8). This tendency is exaggerated by the relatively low thermal conductivity of the plagioclase-like discs compared to the quartz-like matrix (cf. Table 2). The temperature gradients and the significant deviation of thermal expansion coefficients between matrix and discs lead to high maximum principal stresses (Fig. 9, Fig. 10). These are expected to cause interface damage of the discs in a wide range around the irradiation spot.

**Fig. 13:**
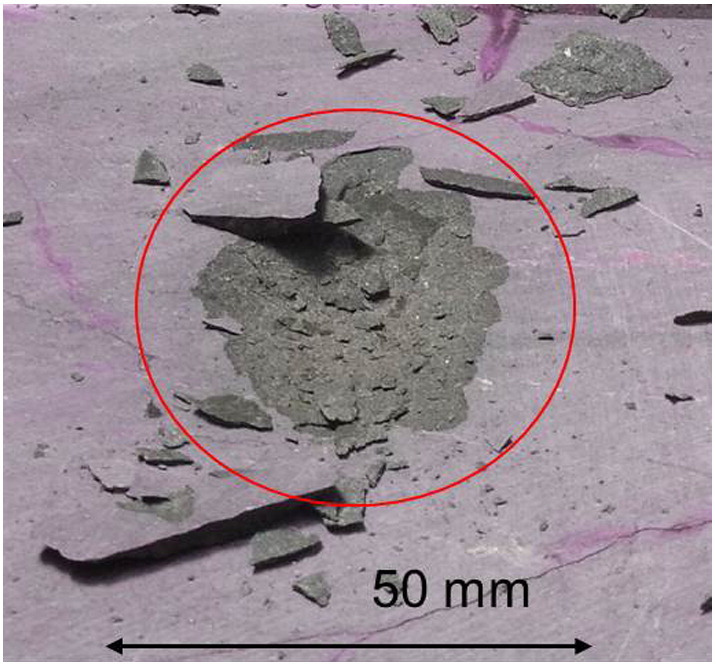
Spot on basalt irradiated with 2.45 GHz microwaves for 15 s. The power of the microwave source is 25 kW. Losses are estimated to be 30% yielding 17.5 kW available for absorption in the rock. Spallation is clearly visible. The irradiated area has approximately the size of the circle.

**Fig. 14:**
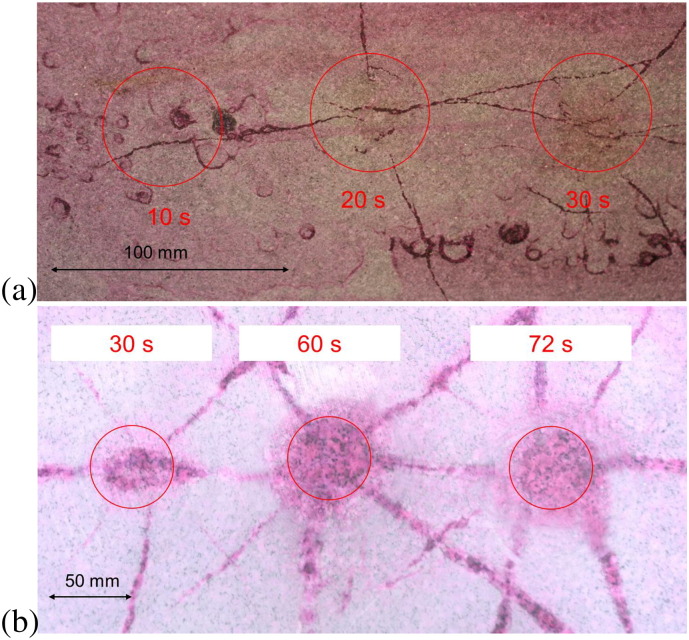
Three spots (indicated by red circles) on gabbro (a) and granite (b) after irradiation with 25 kW (power of the microwave source) for durations given in the figures. Cracks develop due to irradiation for more than 30 and 20 s, respectively, and extend far outside the irradiated area. Surface treated with penetration spray to provide better visibility of cracks.

In order to validate the numerical models qualitatively preliminary experiments on basalt, gabbro and granite were performed at 2.45 GHz (Fig. 13, Fig. 14, respectively). A block of rock (50 · 50 · 30 cm^3^, 30 cm in the direction of microwave propagation) was irradiated using an open-ended rectangular waveguide as the applicator. Its opening was 4.3 · 8.6 cm^2^. Due to the field distribution in the waveguide and a few centimeters distance between waveguide and rock the area of the irradiated spot on the rock is rounded. The region of high intensity is approximately shown by a circle with 5 cm diameter. The figures show examples of spallation of basalt and cracking of gabbro and granite. This indicates that both types of damage can occur but that the details depend on the type of rock, in particular on its thermal and mechanical properties.

The discussion shows that our analysis – the full chain from the microwave irradiation to the stress distribution – includes novel concepts extending the results of previous work (Wang et al., 2008, Wang and Djordjevic, 2014, Whittles et al., 2003) where the microwave propagation in the rock was not included and the absorption was simplified by a pre-defined heat flux. Summarizing our results, we note that gradients – primarily occurring in the distribution of the absorbed microwave power – are essential for the build-up of stresses, however, that the existence of material inhomogeneities considerably increases the strength of the gradients and the probability of damage. The maximum values of the major principal stress, being the relevant failure measure in brittle materials, occur at the grain–matrix interface indicating the onset of possible interface debonding as crack initiation mechanism. Microwave absorption is influenced by the size of the irradiated block of rocks or lumps of ore. The heating behavior depends on the relation of penetration depth *D*_*p*_ of the microwave and size as well as heterogeneity of the irradiated material. The results presented show how quasi-heterogeneous rocks with *D*_*p*_ being smaller than and of the order of the block size are heated and damaged with microwaves. For situations where *D*_*p*_ > block size even in quasi homogeneous rocks temperature gradients are generated due to heat transfer to the surrounding medium. Differences in the dielectric properties between matrix and absorbing constituent in heterogeneous material can nevertheless cause strong inhomogeneous heating. This applies, e.g., to disseminated lumps of ores in mineral processing applications. Having strongly inhomogeneous dielectric and thermo-mechanical properties they are deduced well suitable for microwave treatment also under the condition *D*_*p*_ > lump size.

Our thermo-mechanical analysis considered an extreme case assuming two phases where one phase is transparent for microwaves and the stresses occur at the grain–matrix interfaces. This case is encountered in certain rocks and ores. However, many hard rocks show weaker differences in the permittivity and hence lower stresses are expected. Nevertheless, significant damage can also occur if rocks considered as quasi-homogeneous, such as basalt, are treated with microwaves. In this case other driving forces for damage are crucial such as the thermal conductance at the boundaries or the gradients of the intensity of the microwave beam.

## 6 Conclusions

As an effect of the scattering by the discs deviations of the local *E*_*y*_^2^ values for the heterogeneous model from the homogeneous model are up to 10% for cases with medium to strong absorption in the discs (up to 20% without absorption) in the region of the main microwave beam. Also very weak microwave fields appear far outside the main beam. For a rough approximation to the actual field distribution and for “slow heating” (see below) a Gaussian beam can be assumed for most known rocks (without metallic constituents) and the calculation can be performed with the effective values of the permittivity. For accurate calculations, particularly for “fast heating”, the actual distribution must be taken into account.

Regarding absorbed energy our two-component model assumes a limiting case, viz. that energy is absorbed in the discs (*κ*_*d*,*i*_ ≠ 0) only (the extension to absorption also in the matrix is straightforward). This leads to strong selective heating. For comparison with experimental situations two cases have to be distinguished. (a) “Fast heating” (= selective heating) on time scales shorter than the time for heat conduction between the heated discs (or the absorbing constituents in a real rock). In this case temperature gradients and induced stresses will occur on short distances. Their calculation will have to take into account the heterogeneity of the rock. (b) “Slow heating” allows for the heat conduction in the course of the irradiation. Then, temperature gradients will develop on distances of the width of the beam and the penetration depth of the microwave. Highest stresses are expected to occur in the regions of strongest temperature gradients. This scenario will particularly apply to a quasi-homogeneous rock like basalt. In rocks with strong heterogeneity, even for a smooth distribution of the microwave field and the temperature, stresses can have short-range variations due to the different thermal expansion of the constituents.

Whatever the details of the build-up of stresses are, if the stress exceeds the strength of the rock damage occurs, e.g. spallation or cracks that can even extend over a larger volume than the primarily irradiated one. For the case of “slow heating” this conclusion is supported by preliminary experiments on basalt, gabbro and granite (Fig. 13, Fig. 14, respectively).

The results of the thermo-mechanical analyses (Fig. 8, Fig. 9, Fig. 10) lead to the conclusion that inhomogeneous thermal and mechanical material properties combined with selective absorption are essential for enhanced damage due to the microwave treatment. Damage like cracks and spallation will lead to significantly reduced mechanical forces required for the fragmentation of rocks and ores in mining and mineral processing.

This work shows that the heterogeneous nature of rocks has to be considered for understanding microwave induced stress and damage. In further investigations the actual microstructure and the orientation of the grains will be taken into account in order to obtain a deeper understanding of the stress distributions.

## Figures and Tables

**Table 1:** Parameters of the model rock.

Lattice constant *a*	0.71 cm
Disc diameter	0.467 cm
Filling factor *f*	0.34
Disorder, max. *δ*	25% of lattice constant *a*
Size of model rock	50 cm · 30 cm

**Table 2:**
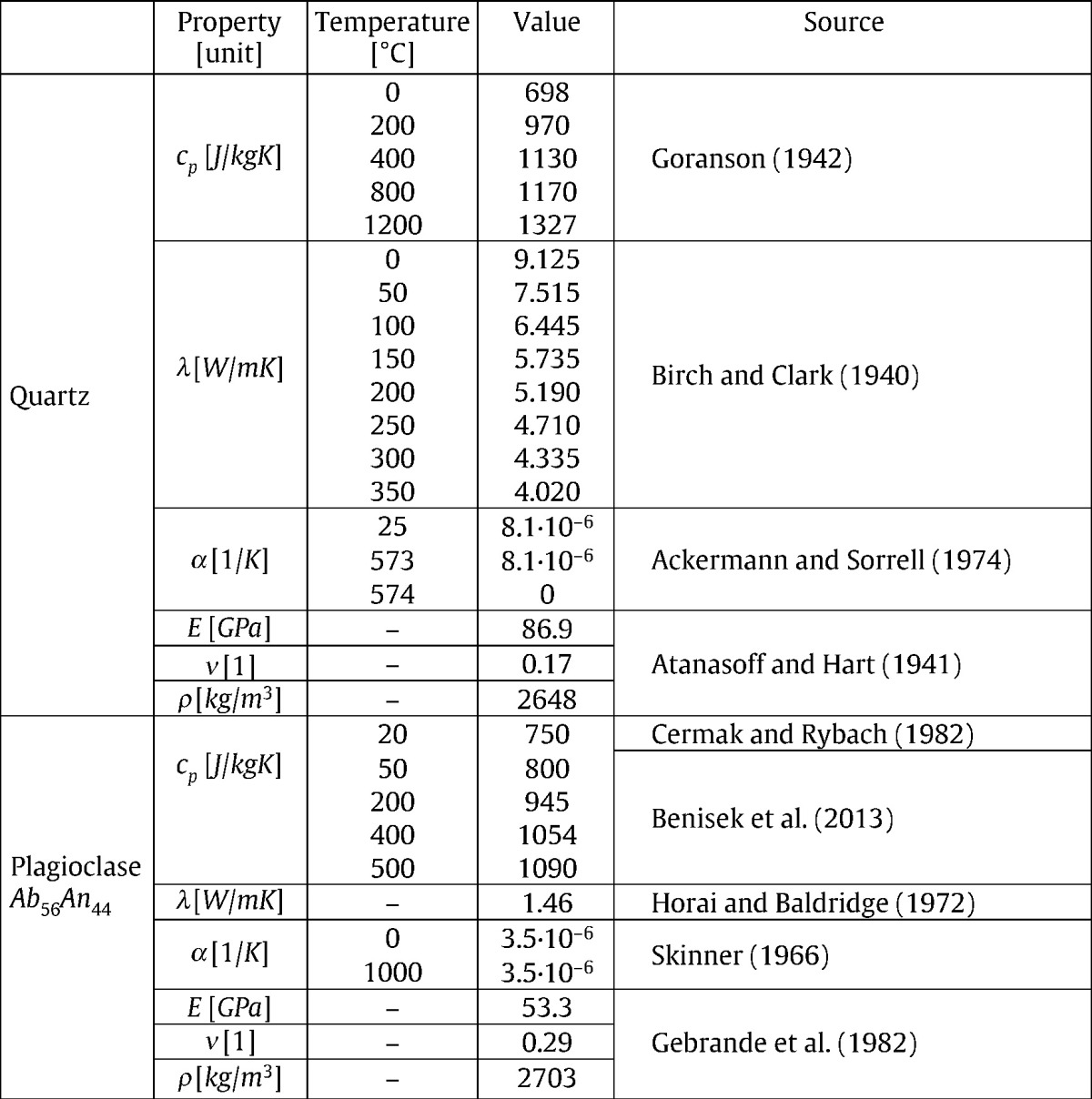
Temperature dependent thermo-mechanical properties of quartz and plagioclase. *c*_*p*_ = specific heat, *λ* = thermal conductivity, *α* = thermal expansion coefficient, *E* = Young's modulus, *ν* = Poisson's ratio, and *ρ* = density. Goranson, 1942, Birch and Clark, 1940, Ackermann and Sorrell, 1974, Atanasoff and Hart, 1941, Cermak and Rybach, 1982, Benisek et al., 2013, Horai and Baldridge, 1972, Skinner, 1966, Gebrande et al., 1982.
